# Supplementary material for: Effect of Terahertz Electromagnetic Field on the Permeability of Potassium Channel Kv1.2
Source: Int J Mol Sci. 2023 Jun 17;24(12):10271. doi: 10.3390/ijms241210271 (PMC10299135; doi:10.3390/ijms241210271)
Supplement: Supplementary file 1 [file ijms-24-10271-s001.zip › ijms-2379806-supplementary.pdf]

Supplement Data

Effect of terahertz electromagnetic field on the permeability of potassium channel Kv1.2

Wen Ding [1,2,†], Xiaofei Zhao [1,2,†], Hongguang Wang [1,2], Yize Wang [1,2], Yanjiang Liu [1,2], Lirong Gong [1,2], Shu Lin [1,2], Chunliang Liu [1,2,*] and Yongdong Li [1,2,*]

[1] Key Laboratory for Physical Electronics and Devices of the Ministry of Education, Xi'an Jiaotong University, Xi'an 710049, China; dingwen@mail.xjtu.edu.cn (W.D.); zhaoxiaofei@stu.xjtu.edu.cn (X.Z.); wanghg@mail.xjtu.edu.cn (H.W.); wangyize@stu.xjtu.edu.cn (Y.W.); yanjiang@stu.xjtu.edu.cn (Y.L.); gonglr@stu.xjtu.edu.cn (L.G.); shulin@mail.xjtu.edu.cn (S.L.)

[2] School of Electronic Science and Engineering, Xi'an Jiaotong University, Xi'an 710049, China

* Correspondence: chlliu@mail.xjtu.edu.cn (C.L.); leyond@mail.xjtu.edu.cn (Y.L.);
Tel.: +86-15929942829 (C.L.); +86-029-82668657 (Y.L.)

† These authors contributed equally to this work.

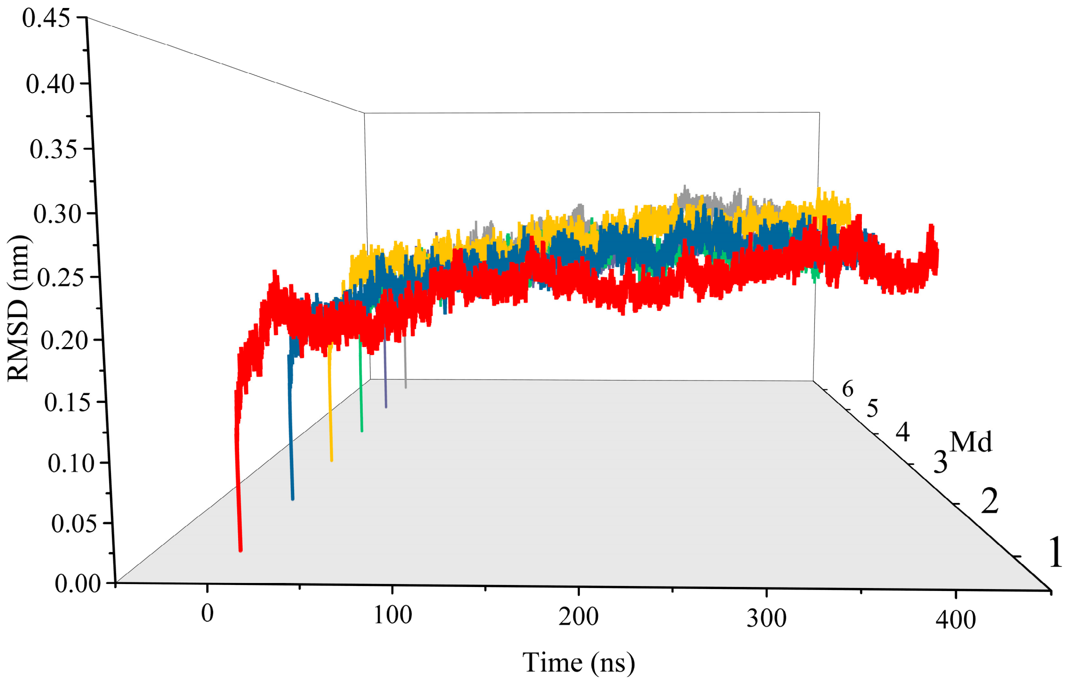

Figure S1: Root Mean Square Deviation (RMSD) of the channel without an applied electric field.

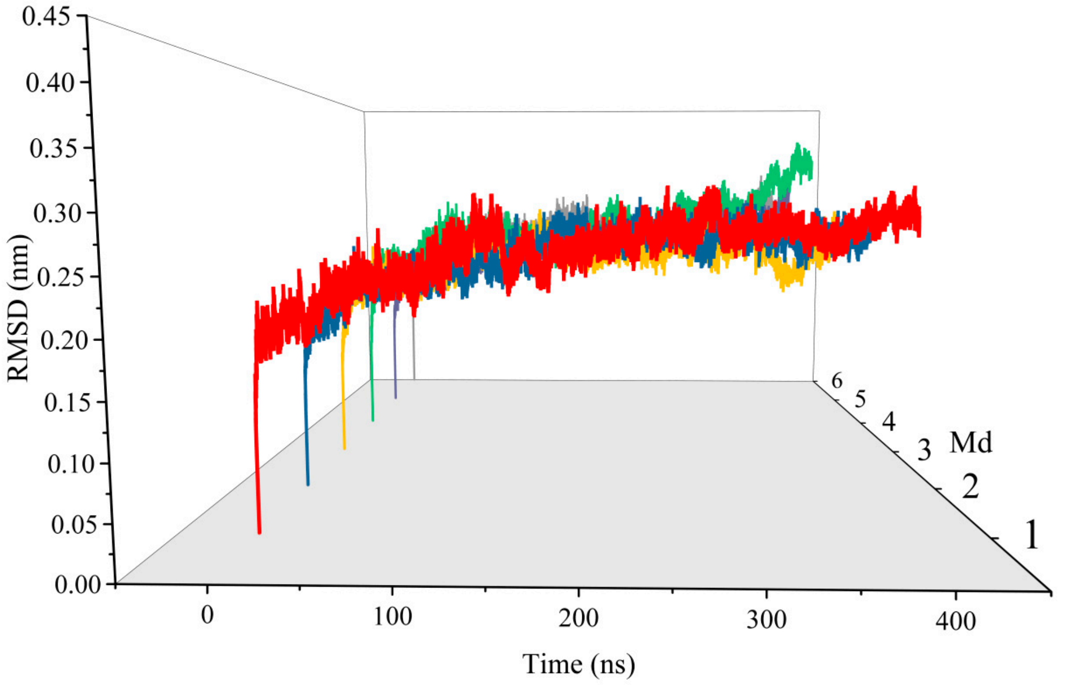

Figure S2: Root Mean Square Deviation (RMSD) of the channel with an applied electric field of 4THz.

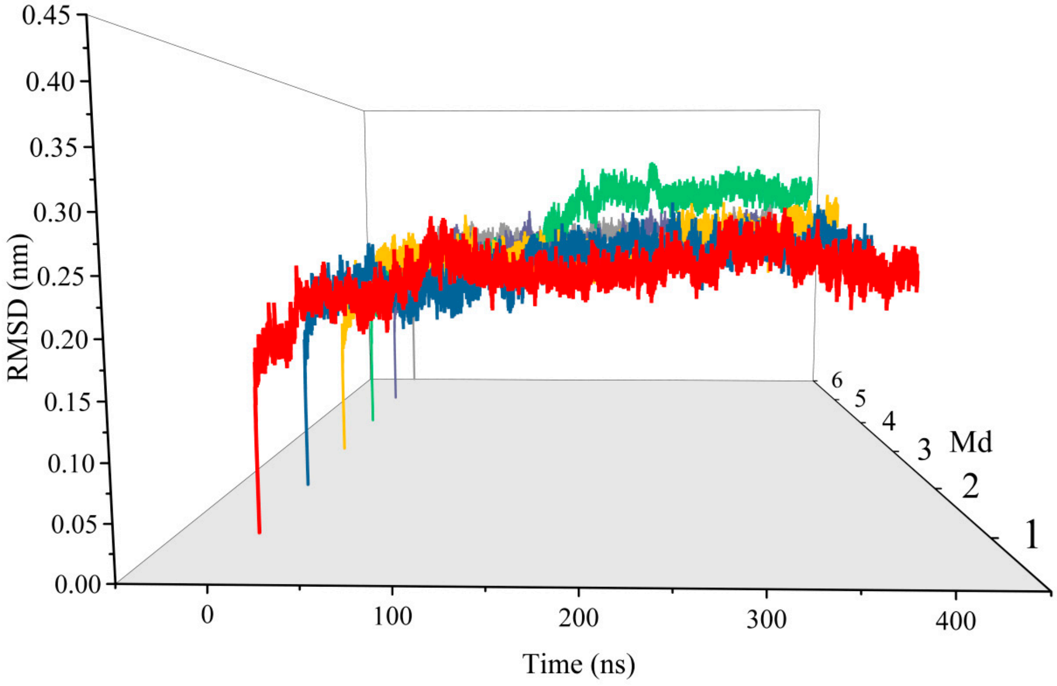

Figure S3: Root Mean Square Deviation (RMSD) of the channel with an applied electric field of 10THz.

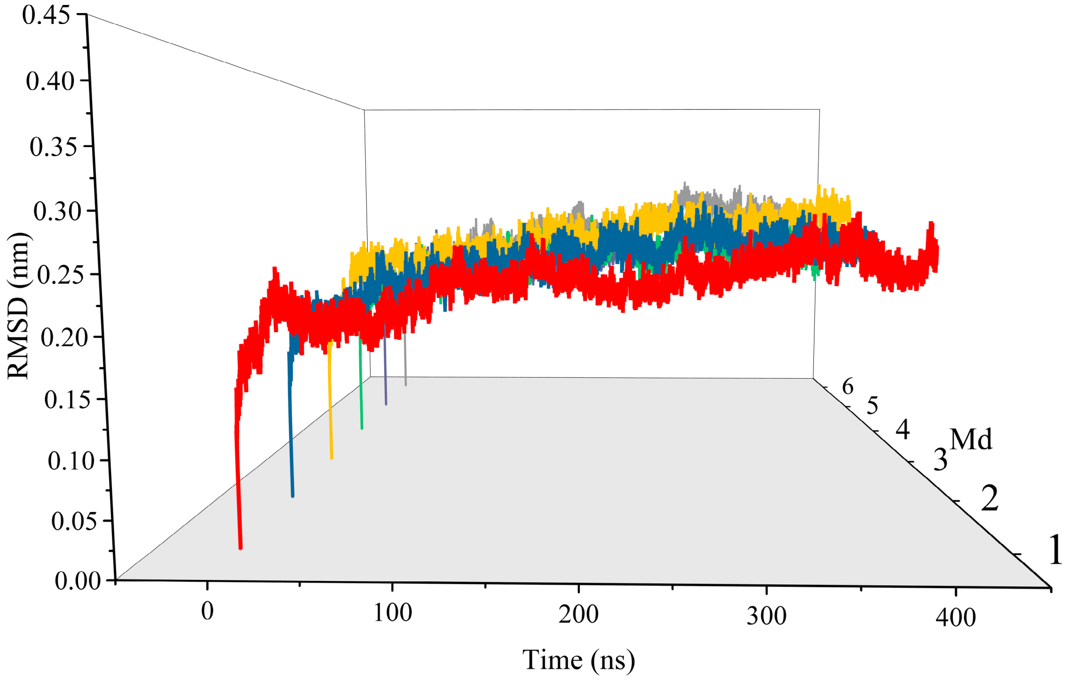

Figure S4: Root Mean Square Deviation (RMSD) of the channel with an applied electric field of 15THz.

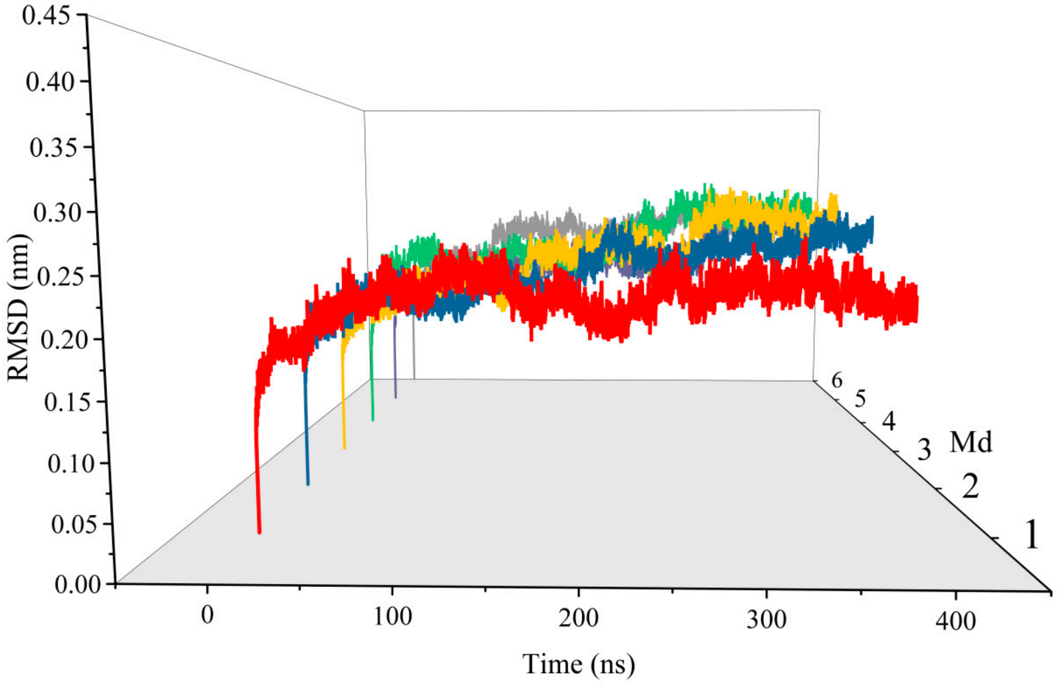

Figure S5: Root Mean Square Deviation (RMSD) of the channel with an applied electric field of 20THz.
